# A Multicenter Assessment of Anatomic Suitability for Iliac Branched Devices in Eastern Asian Patients With Unilateral and Bilateral Aortoiliac Aneurysms

**DOI:** 10.3389/fcvm.2021.763351

**Published:** 2022-01-03

**Authors:** Zheyun Li, Min Zhou, Guili Wang, Tong Yuan, Enci Wang, Yufei Zhao, Xiaolong Shu, Yuchong Zhang, Peng Lin, Weiguo Fu, Lixin Wang

**Affiliations:** ^1^Department of Vascular Surgery, Zhongshan Hospital, Fudan University, Shanghai, China; ^2^Vascular Surgery Institute of Fudan University, Shanghai, China; ^3^Department of Vascular Surgery, Nanjing Drum Tower Hospital, Nanjing University Medical School, Nanjing, China; ^4^Department of Vascular Surgery, Affiliated Jinan Central Hospital of Shandong First Medical University, Jinan, China; ^5^Department of Vascular Surgery, Xiamen Branch, Zhongshan Hospital, Fudan University, Xiamen, China

**Keywords:** abdominal aortic aneurysm (AAA), aortoiliac aneurysm (AIA), iliac branch device (IBD), bilateral lesions, Eastern Asian patients

## Abstract

**Objective:** This study aims to assess the suitability of four types of commercial iliac branch device systems to treat Eastern Asian abdominal aortic aneurysm (AAA) patients with bilateral or unilateral common iliac artery aneurysms (CIAAs).

**Methods:** Patients with a coexisting AAA and a unilateral or bilateral CIAAs who underwent endovascular aneurysm repair (EVAR) at two tertiary centers in China from 2015 to 2017 were reviewed. Morphology of lesions was measured and the anatomic suitability for Cook iliac branch device (IBD), Gore iliac branch endoprosthesis (IBE), Lifetech iliac branch stent graft (IBSG), and Jotec IBD was evaluated according to the latest instructions for use.

**Results:** Seventy-six patients with AAA were enrolled, including 35 bilateral CIAAs, 41 unilateral CIAAs. A hundred and eleven lesions were investigated aggregately: 16.2, 28.8, 21.6, and 19.8% met the criteria for Cook IBD, Gore IBE, Lifetech IBSG, and Jotec IBD, respectively. A total of 34 (44.7%) patients could be treated for at least one lateral lesion. The diameter of the internal iliac artery (IIA) was the most common restriction for IBD application. Additionally, the IIA diameter of lesions in the bilateral group was significantly larger compared with the unilateral group (*P* < 0.001). Based on the anatomical characteristics alone, it is likely that IBDs will be more suitable for unilateral lesions than bilateral ones (*P* < 0.05). However, there was no difference between the suitability for patients with unilateral or bilateral CIAAs (*P* > 0.05).

**Conclusions:** Less than half of Eastern Asian patients with aortoiliac aneurysms were eligible for IBD application. This was primarily due to the IIA diameter failing to meet the criteria. And thus, the suitability of lesions in bilateral group was significantly lower than that in the unilateral group. Aiming to expand the indications and optimize the design of the iliac branch devices, IIA diameter and the anatomical characteristics of the bilateral lesions should be considered deliberately.

## Introduction

Aneurysmal degeneration of the iliac arteries can occur alone or in association with other large vessel aneurysms. Approximately 15-40% of patients presenting with an abdominal aortic aneurysm (AAA) also have a concomitant unilateral or bilateral common iliac artery aneurysm (CIAA) (1–3). For patients with a coexisting CIAA and AAA undergoing endovascular aneurysm repair (EVAR), it is essential to provide adequate distal fixation of the iliac limb into the iliac artery. Nevertheless, the complex anatomical characteristics of the CIAA pose a challenge for device implantation, distal anchoring, and complete aneurysm exclusion. There is evidence indicating that not all patients fit the instructions for use (IFUs) of standard bifurcated endografts (4). A common approach is to extend the limb directly into the external iliac artery (EIA), with or without concomitant internal iliac artery (IIA) embolization (5–7). However, sacrificing both IIAs may result in buttock claudication, erectile dysfunction, colonic ischemia, and spinal cord ischemia (8–10). Moreover, ischemic complications are observed in 30-55% of the patients even after unilateral IIA occlusion (11).

In order to prevent these potential complications, iliac branch devices (IBD) have been developed to preserve perfusion through unilateral or bilateral hypogastric arteries when excluding CIAA. Three commercial IBD configurations by respective manufacturers, namely, Cook Medical, W. L. Gore & Associates, and Jotec have been developed so far in Western countries, and one designed by Lifetech for Asian patients (6, 12–14). Among those, Lifetech iliac branch stent graft (IBSG) is the only device obtained the China National Medical Products Administration (NMPA) approval. Anatomical characteristics of aortoiliac aneurysm (AIA) have been regarded as a major factor affecting the application of IBDs (4, 5). Therefore, in this study, four types of IBDs were assessed for their suitability of Eastern Asian patients with AIA according to their instruction for use (IFU). Moreover, since bilateral implantation of IBDs is considered to be a safe and effective technique to preserve antegrade IIA flow and help decrease potential ischemic complications further (15), the suitability of unilateral and bilateral lesions was also taken into account. An appropriate choice of IBDs to treat Eastern Asian patients is intended to be provided to vascular surgeons through this study, as well as suggestions to improve future generations of iliac branch technologies.

## Materials and Methods

### Study Design and Population

This retrospective, multicenter study was performed in accordance with the principles of the Declaration of Helsinki and approved by the Institutional Review Board of Zhongshan Hospital, Shanghai (approval no. B2018-045) and Drum Tower Hospital, Nanjing (approval no. 2017-015-05). The retrospective data were anonymous, and the requirement for informed consent was therefore waived. Both of these international vascular centers receive patients predominantly from China and East Asia. A total of 1,049 patients received EVAR and 119 underwent open repair in the two institutions between 2015 and 2017. We totally reviewed 76 patients with an infrarenal AAA and coexisting CIAA (35 patients with bilateral CIAAs, 41 patients with a unilateral CIAA) who underwent EVAR from 2015 to 2017. A common iliac artery aneurysm ≥ 2.5 cm concomitant with AAA would be treated simultaneously during EVAR. The threshold to include patients in this study cohort was the presence of a unilateral or bilateral CIA of at least 25 mm in diameter associated with a concomitant AAA. All patients who underwent repair for aneurysm rupture, pseudoaneurysm, solitary CIA aneurysm, or mycotic aneurysm were excluded from this study.

### Anatomic Measurement

Preoperative computed tomography angiography (CTA) images of the aortoiliac from the enrolled patients were obtained. All imaging data were reviewed on a three-dimensional workstation using Vitrea fX software (Vital Images, Minnetonka, MN, USA). Briefly, a centerline was generated in the aorta from the infrarenal aorta to the bilateral EIAs and IIAs, and aortoiliac lengths and diameters were measured based on centerline images. The maximum diameter of the artery was measured from the adventitia and the mural thrombus was considered as well. We compared the length of EIA with relative IBD criteria rather than presenting the exact measured value directly. We also added annotations on those with poor vascular condition such as severe occlusion, stenosis, calcification, and inappropriate bifurcation angle of iliac artery. A vascular surgeon with at least 3 years of experience and a radiology attending physician performed all measurements independently.

### Iliac Branch Device Systems

Currently, there are only four types of iliac branch device systems off the shelf worldwide, which were designed by Cook Medical (Bloomington, IN, USA), W. L. Gore & Associates (Flagstaff, AZ, USA), Lifetech (Shenzhen, Guangdong, China), and Jotec (Hechingen, BW, Germany). The components of respective IBD systems were taken into consideration as well. Among all IBDs, only Lifetech IBSG is approved in China. The exclusion criteria of these devices are described in Table 1 according to the latest IFUs.

**Table 1:** Summary of exclusions for the four IBDs based on anatomical characteristics.

	**Cook IBD**	**Gore IBE**	**Lifetech IBSG**	**Jotec IBD**
CIA length (mm)	<50	<40 or Aortoiliac length <165	<40	<40
CIA diameter (mm)	<16	<17	<18	<18
EIA length (mm)	<20	<10	<15	<15
EIA diameter (mm)	<8 or >11	<6.5 or >25	<8.4 or >14.5	<8 or >13
IIA length (mm)	<10	<10	<10	<15
IIA diameter (mm)	<6 or >11.4	<6.5 or >13.5	<5 or >11.4	<6 or >11.4

### Statistical Analysis

Based on the IFU requirements, we evaluated the anatomical features of lesions to determine if each device was suitable. Accordingly, IBD applicability for patients was assessed in terms of lesion suitability. Descriptive statistics were presented as mean with standard deviation or median with range. The Chi-square test was used to compare the applicability of four IBDs. A *P*-value <0.05 was considered statistically significant. Calculation and comparison of all data were performed in Excel (Microsoft, Redmond, WA, USA) and SPSS 25.0 (IBM Corp., Armonk, NY, USA).

## Results

From 2015 to 2017, 76 AAA patients with 41 unilateral and 35 bilateral CIAAs were identified in Zhongshan Hospital and Nanjing Drum Tower Hospital. The IIA maximum diameters were measured at 18.3 ± 5.1 and 15.3 ± 7.9 mm on the left and right side, while the EIA max diameters were 11.2 ± 2.2 and 11.3 ± 2.9 mm, respectively. The average IIA max diameter in patients with bilateral lesions (18.6 ± 8.9 mm) was larger than those with unilateral lesions (12.9 ± 6.4 mm, *P* < 0.001). The anatomical characteristics of lesions are summarized in Table 2. Lesions in the unilateral group exhibited a significant anatomical difference from lesions in the bilateral group (Table 3).

**Table 2:** Anatomical characteristics of aortoiliac aneurysms of Eastern Asian patients (mm).

	**Zhongshan cohort (*****n*** **=** **56)**			**Nanjing cohort (*****n*** **=** **55)**			**All (*****n*** **=** **111)**		
	**Mean**	**SD**	**Median (range)**	**Mean**	**SD**	**Median (range)**	**Mean**	**SD**	**Median (range)**
Left CIA max. diameter	30.0	5.2	28.7 (25.0–45.8)	34.6	8.8	32.3 (25.1–51.9)	32.6	7.7	30.7 (25.0–51.9)
Right CIA max. diameter	32.3	8.4	31.0 (25.0–66.0)	34.3	11.2	30.8 (25.1–67.2)	33.2	9.7	30.9 (25.0–67.2)
Left CIA length	55.1	16.3	52.5 (30.0–88.0)	61.3	20.8	63.7 (24.5–94.5)	58.6	19.0	56.6 (24.5–94.5)
Right CIA length	56.7	16.2	58.3 (23.0–92.0)	61.6	22.1	61.5 (22.2–111.4)	58.9	19.1	59.0 (22.2–111.4)
Left IIA max. diameter	16.6	9.1	16.4 (5.1–35.9)	19.5	8.8	18.6 (8.4–45.1)	18.3	5.1	18.3 (5.1–45.1)
Right IIA max. diameter	13.0	8.5	10.5 (4.9–41.0)	18.1	6.2	17.4 (7.8–31.4)	15.3	7.9	13.9 (4.9–41.0)
Left IIA length	51.3	10.1	50.1 (33.9–74.3)	49.0	14.3	45.4 (28.6–76.7)	50.0	12.6	47.0 (28.6–76.7)
Right IIA length	53.5	8.6	52.6 (39.6–73.3)	51.1	11.3	49.2 (27.3–77.7)	52.4	9.9	52.0 (27.3–77.7)
Left EIA max. diameter	9.9	1.4	9.9 (7.5–13.4)	12.2	2.2	12.2 (9.1–19.3)	11.2	2.2	10.7 (7.5–19.3)
Right EIA max. diameter	10.2	1.6	10.0 (7.7–14.0)	12.7	3.4	11.8 (8.8–25.1)	11.3	2.9	10.8 (7.7–25.1)

**Table 3:** Comparison of anatomical characteristics for unilateral and bilateral lesions (mm).

	**Unilateral (*n* = 41)**	**Bilateral (*n* = 35)**	***P*-value**
CIA max. diameter	30.7 ± 5.3	34.3 ± 10.3	0.016
CIA length	54.0 ± 14.9	61.6 ± 20.6	0.029
IIA max. diameter	12.9 ± 6.4	18.6 ± 8.9	<0.001
IIA length	52.4 ± 10.5	50.9 ± 11.4	0.477
EIA max. diameter	10.6 ± 1.6	11.6 ± 3.0	0.022

**Figure 1:**
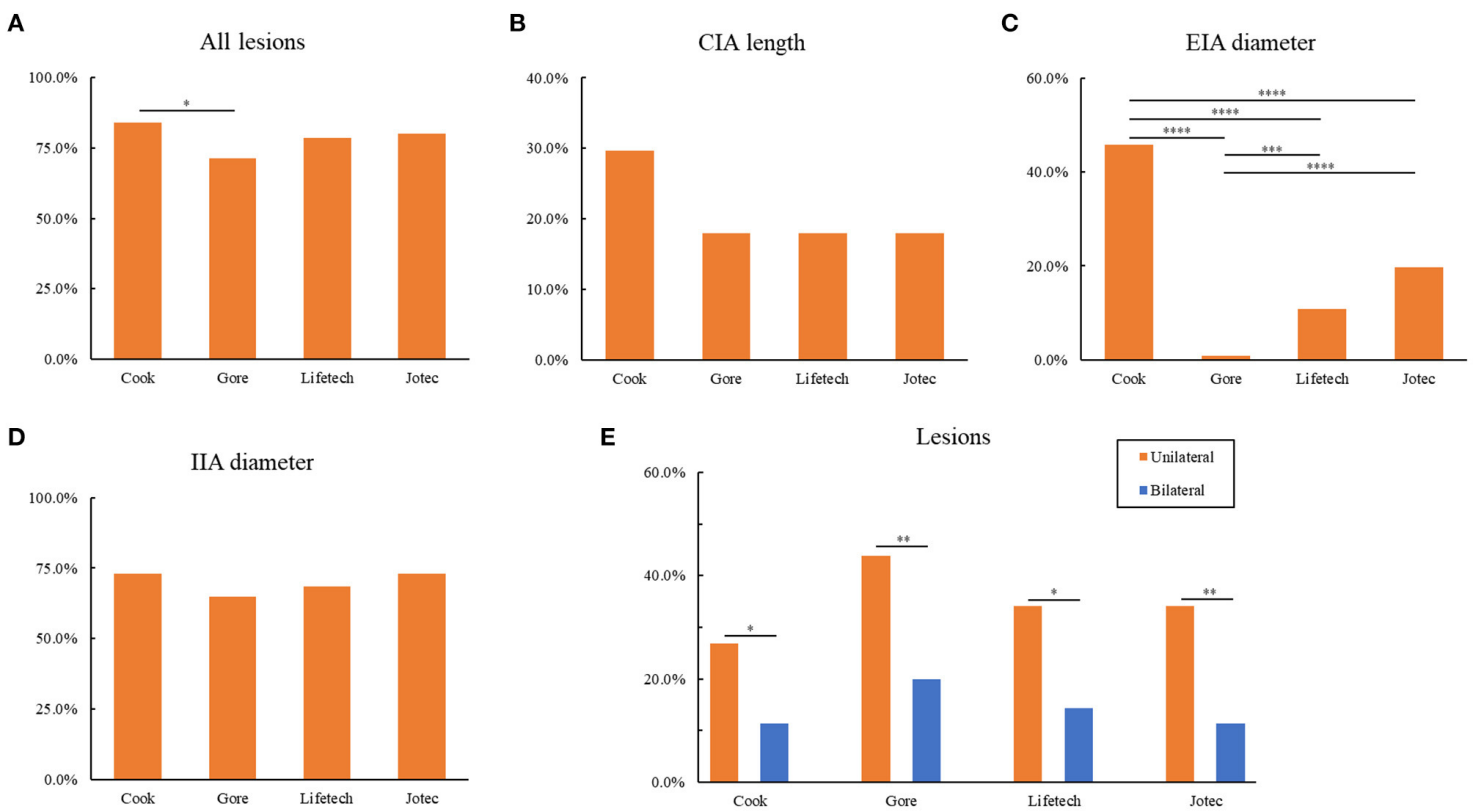
Exclusion for IBDs based on anatomical characteristics. **(A)** Exclusion of all lesions for four IBDs. **(B)** Exclusion of CIA length for four IBDs. **(C)** Exclusion of EIA diameter for four IBDs. **(D)** Exclusion of IIA diameter for four IBDs. **(E)** Comparison of the exclusion of unilateral and bilateral lesions for four IBDs. *P < 0.05; **P < 0.01; ***P < 0.001; ****P < 0.0001; as determined by Chi-square test. IBD, iliac branch device; CIA, common iliac artery; EIA, external iliac artery; IIA, internal iliac artery.

Based on the anatomical characteristics, the suitability of the 111 unilateral and bilateral pathological CIAs for the four types of IBDs are depicted in Table 4. By strictly evaluating the applicability to each lesion, 74 (66.7%) lesions failed to meet the required criteria of any IBD. Specifically, 93 lesions (83.8%) were excluded based on Cook's criteria, which was significantly higher than the number excluded by Gore's (79, 71.2%, Figure 1A). As for Lifetech and Jotec, 87 (78.4%) and 89 (80.2%) did not meet the criteria, respectively. IIA diameter was the most common reason for the exclusion of all four IBDs. Nearly three-quarters of the lesions (81, 73.0%) were excluded by Cook criteria due to IIA diameter, 72 (64.9%) by Gore, 76 (68.5%) by Lifetech and, 81 (73.0%) by Jotec. Another major criterion for exclusion was CIA length. Thirty-three (29.7%) lesions were excluded by Cook and 20 (18.0%) by the other three IBDs. Nevertheless, there is no statistical difference in CIA length or IIA diameter exclusion rate among the four IBDs (Figures 1B,D). However, IBDs showed significant differences in the exclusion rate caused by the EIA diameter (Figure 1C). In addition, IBD applicability of lesions in the unilateral group was significantly higher than that in the bilateral group (*P* < 0.05, Figure 1E), which is mainly because of the difference in IIA diameter (Table 4).

**Table 4:** Anatomic reasons for exclusion and comparison for unilateral and bilateral lesions.

**Exclusion criteria**	**Lesion excluded**			
	**Unilateral (*n* = 41)**	**Bilateral (*n* = 70)**	***P*-value**	**All (*n* = 111)**
**ALL**	21 (51.2%)	53 (75.7%)	0.008	74 (66.7%)
**Cook IBD**	30 (73.2%)	62 (88.6%)	0.036	93 (83.8%)
CIA length <50 mm	14 (34.1%)	19 (27.1%)	0.285	33 (29.7%)
CIA diameter <16 mm	0 (0%)	0 (0%)	–	0 (0%)
EIA length <20 mm	0 (0%)	0 (0%)	–	0 (0%)
EIA diameter <8 or > 11 mm	17 (41.5%)	34 (48.6%)	0.299	51 (45.9%)
IIA length <10 mm	0 (0%)	0 (0%)	–	0 (0%)
IIA diameter <6 or > 11.4 mm	22 (53.7%)	59 (84.3%)	0.001	81 (73.0%)
**Gore IBE**	23 (56.1%)	56 (80.0%)	0.007	79 (71.2%)
Aortoiliac length <165 or CIA length <40 mm	7 (17.1%)	13 (18.6%)	0.529	20 (18.0%)
CIA diameter <17 mm	0 (0%)	0 (0%)	–	0 (0%)
EIA length <10 mm	0 (0%)	0 (0%)	–	0 (0%)
EIA diameter <6.5 or > 25 mm	0 (0%)	1 (1.4%)	0.631	1 (.9%)
IIA length <10 mm	0 (0%)	0 (0%)	–	0 (0%)
IIA diameter <6.5 or > 13.5 mm	20 (48.8%)	52 (74.3%)	0.006	72 (64.9%)
**Lifetech IBSG**	27 (65.9%)	60 (85.7%)	0.014	87 (78.4%)
CIA length <40 mm	7 (17.1%)	13 (18.6%)	0.529	20 (18.0%)
CIA diameter <18 mm	0 (0%)	0 (0%)	–	0 (0%)
EIA length <15 mm	0 (0%)	0 (0%)	–	0 (0%)
EIA diameter <8.4 or > 14.5 mm	3 (7.3%)	9 (12.9%)	0.283	12 (10.8%)
IIA length <10 mm	0 (0%)	0 (0%)	–	0 (0%)
IIA diameter <5 or > 11.4 mm	20 (48.8%)	56 (80.0%)	0.001	76 (68.5%)
**Jotec IBD**	27 (65.9%)	62 (88.6%)	0.004	89 (80.2%)
CIA length <40 mm	7 (17.1%)	13 (18.6%)	0.529	20 (18.0%)
CIA diameter <18 mm	0 (0%)	0 (0%)	–	0 (0%)
EIA length <15 mm	0 (0%)	0 (0%)	–	0 (0%)
EIA diameter <8 or > 13 mm	4 (9.8%)	18 (25.7%)	0.033	22 (19.8%)
IIA length <15 mm	0 (0%)	0 (0%)	–	0 (0%)
IIA diameter <6 or > 11.4 mm	22 (53.7%)	59 (84.3%)	0.001	81 (73.0%)

**Figure 2:**
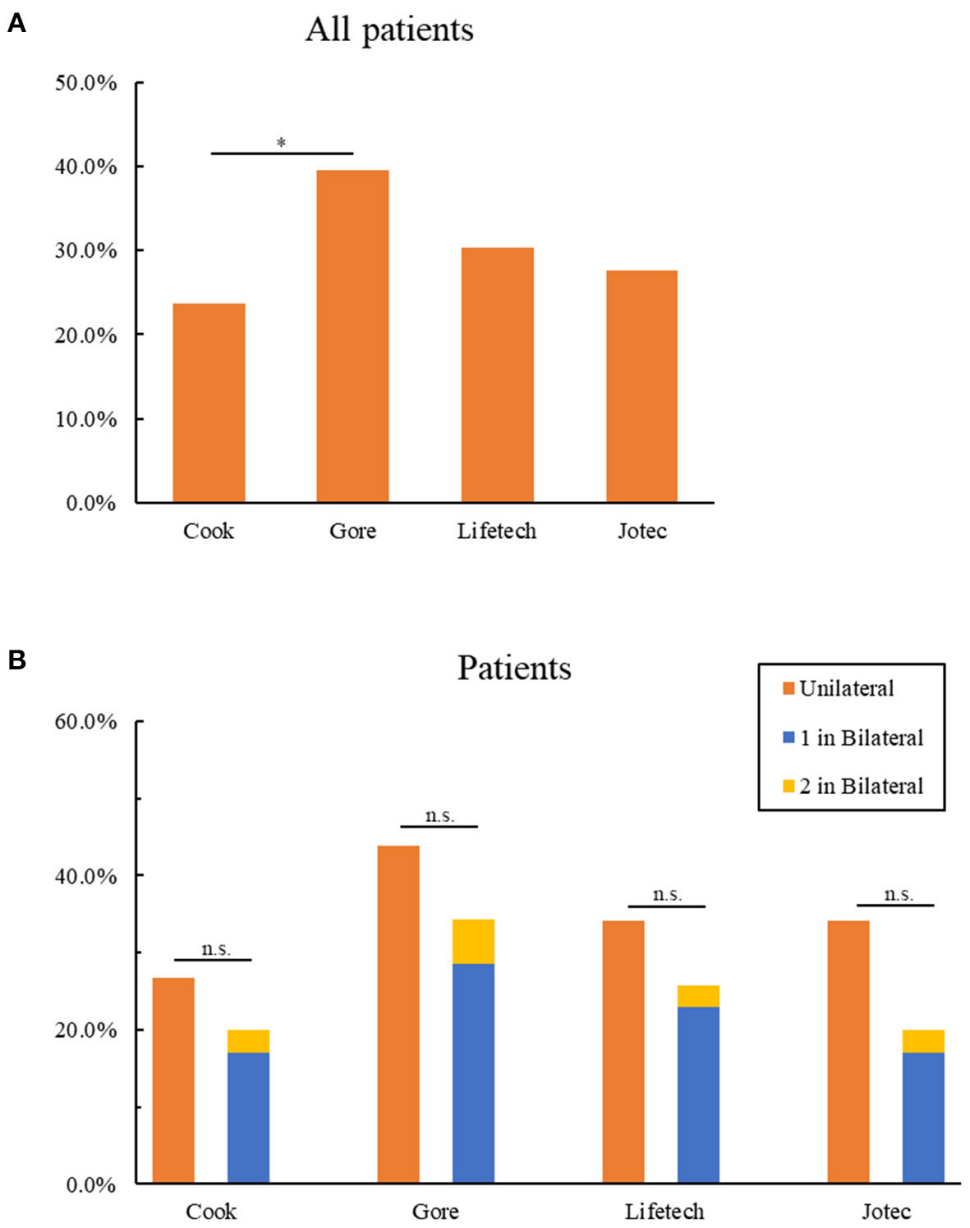
Anatomic suitability of patients for IBDs. **(A)** Exclusion of all patients for four IBDs. **(B)** Comparison of the exclusion of patients with unilateral and bilateral lesions for four IBDs. 1 in bilateral represent patients with bilateral lesions who could be only treated one side by IBDs; 2 in bilateral represent patients with bilateral lesions who could be treated both sides by IBDs *P < 0.05; n.s. *P* > 0.05 (no significance); as determined by Chi-square test. IBD, iliac branch device.

We next sought to assess the suitability per patient. The applicability of all types of iliac branch devices for 76 patients is shown in Table 5. Approximately half of the patients (34, 44.7%) could be treated with preserving at least one IIA. There were 30 (39.3%) patients eligible for Gore IBE, and its applicability is significantly higher than Cook IBD (18, 23.7%, Figure 2A). As for the other two IBDs, 24 (21.6%) patients were eligible for Lifetech and 22 (19.8%) for Jotec devices. Suitability per patient with unilateral aortoiliac aneurysm for all IBDs was found to have no statistical discrepancy compared with those who have bilateral lesions (*P* > 0.05, Figure 2B). Of note, only three patients (3.9%) in the bilateral group could be treated on both sides. One of them met the criteria of Gore IBE for the left lesion and the three other IBDs for the right, while the other two patients were eligible for all IBDs for both sides.

**Table 5:** Exclusion for patients with unilateral and bilateral lesions.

	**Unilateral (*n* = 41)**	**Bilateral (*****n*** **=** **35)**			**All (*n* = 76)**
		**1**	**2**	**all**	
Cook IBD	11 (26.8%)	6 (17.1%)	1 (2.9%)	7 (20.0%)	18 (23.7%)
Gore IBE	18 (43.9%)	10 (28.6%)	2 (5.7%)	12 (34.3%)	30 (39.5%)
Lifetech IBSG	14 (34.1%)	8 (22.9%)	1 (2.9%)	9 (25.7%)	23 (30.3%)
Jotec IBD	14 (34.1%)	6 (17.1%)	1 (2.9%)	7 (20.0%)	21 (27.6%)
Total	20 (48.8%)	11 (31.4%)	3 (8.6%)	14 (40.0%)	34 (44.7%)

**Figure 3:**
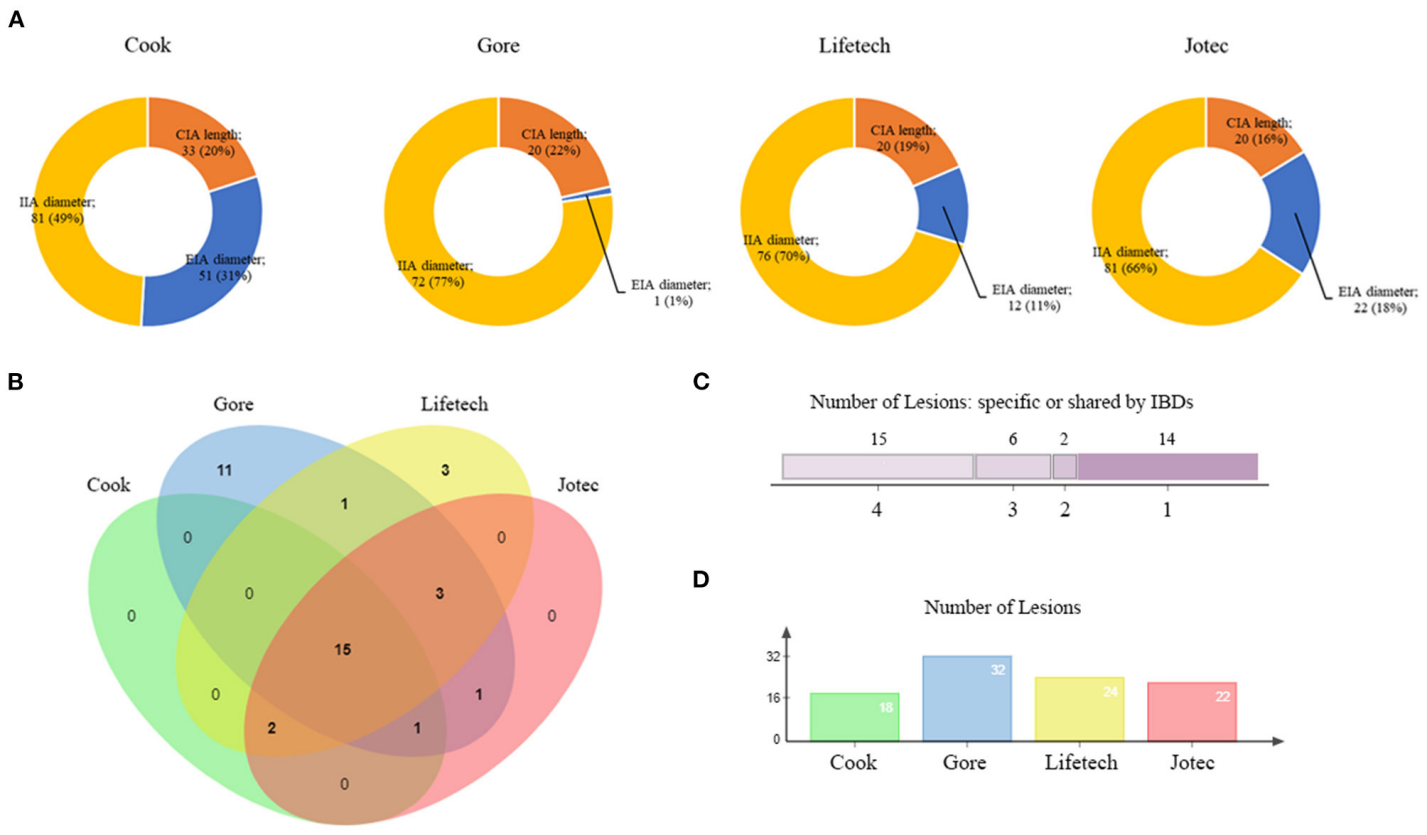
Overview of IBD applicability. **(A)** The proportion of exclusion reasons for four IBDs. **(B)** A four-set Venn diagram showing the numbers of lesions meeting the criteria of IBDs. **(C)** The number of lesions meeting different IBD criteria. **(D)** The number of lesions included by each device. IBD, iliac branch device.

The difference among the four types of IBDs is further analyzed in Figure 3. The diameter of IIA played an essential role in restricting the use of Cook (49%), Gore (77%), Lifetech (70%), and Jotec devices (66%, Figure 3A). The suitability of all the lesions for four IBDs was summarized by a Venn diagram (Figure 3B). Only 15 lesions were eligible for all four types of IBDs. Fourteen lesions met the criteria of merely one device, of which Gore accounted for 11 lesions (Figure 3C). Overall, 32 aortoiliac aneurysms were eligible for Gore IBE which was higher than any other devices (Figure 3D).

## Discussion

Iliac branch devices have been reported as a safe, feasible, and effective solution to preserve IIA blood flow in select patients with suitable anatomy (16, 17). Currently, there are several designs of IBD on clinical trials or commercially available all over the world (18, 19). The devices of Cook and Jotec are undergoing clinical trials in the United States, while Gore IBD has already received Food and Drug Administration approval. IBSG, designed by Lifetech, obtained the market registration approval in China recently. However, the usage of IBDs is limited by anatomical characteristics. Several published literature have analyzed the applicability of Cook and Gore device for AAA patients requiring extension into EIA during EVAR (4, 5). Itoga et al. demonstrated that the anatomic suitability of Japanese patients with aortoiliac aneurysm for those two IBDs was limited by smaller CIA diameter and shorter CIA length (20). Our study analyzes the suitability of four IBDs for Eastern Asian AAA patients with a coexisting CIAA and provides a selecting guidance for physicians who are considering to use any of these IBDs in preservations of IAA for this group of patients.

Seventy-six patients who underwent EVAR were reviewed and all of them present with a unilateral or bilateral CIA of at least 25 mm in diameter associated with a concomitant AAA. We retrospectively measured their morphological features. According to the centerline measurement CTA scans, the average left and right CIA lengths were 56.6 and 59.0 mm, respectively. It was similar to Japanese patients (56.5 mm) reported by Itoga et al. (20), but was significantly shorter than that in American patients (70.8–72 mm) and German patients (68 mm) (5, 21). In addition, Wang et al. measured infrarenal aorta and common iliac artery in Chinese population and found that the normal CIA was approximately 9.7 mm in males and 8.5 mm in females (22). However, the average CIA diameter was 1.2 cm in men and 1.0 cm in women in the United States (23). As with any vessel, a true common iliac artery aneurysm is defined as a focal dilation of the artery with more than 50% in comparison with the normal one (24). Based upon these values, a CIAA is generally present if the artery measures >1.85 cm in males and >1.5 cm in females according to a Western study (24). It indicates that more accurate diagnostic criteria are needed for Asian CIAA patients. Therefore, the standard of Asian CIAA treatment may also change due to the adjustment of diagnostic criteria.

Based on the anatomical characteristics, we assessed the suitability for four types of IBD systems. Unlike the previous studies, it was the first time that the domestic IBSG by Lifetech and newly appeared Jotec devices have been added for evaluation. Moreover, the exclusion criteria were updated in accordance with the latest IFUs. One-third of the lesions (37, 33.3%) would have been eligible for at least one device by pure anatomical criteria. In our study, Gore IBE was found to have a significantly higher applicability than Cook IBD (*P* < 0.05) but no difference with Lifetech or Jotec devices.

Since IAAs often involve the internal and external iliac arteries simultaneously, the dilated arteries might lack a distal sealing zone for IBDs. IIA diameter was found to be the most common exclusion factor for all IBDs in our study. Although Gore IBE had a wide IIA applicability (6.5-13.5 mm), this still resulted in an exclusion rate of 64.9% per lesion. Donas et al. reported that about 34% internal iliac arteries were larger than 12 mm among over 900 IBD implants (25). Simonte et al. also demonstrated a low rate of hypogastric aneurysms in their IBD practice (26). Our study excluded about 70% patients for an IBD implant because of the IIA diameter. Except for those excluded for thin IIAs, there were still roughly 50% of patients ineligible for IBDs owing to a large diameter of IIAs. This may be due to the differences in ethnicity and measurement methods. The devices by different manufacturers showed a difference to some extent in IIA diameter exclusion criteria. It might be due to an internal iliac component contained in Gore and Lifetech IBD systems while Cook and Jotec choose commercial bridging stent such as Advanta V12 (Atrium Medical, Hudson, NH, USA), Viabahn (W.L. Gore &Associates, Flagstaff, AZ, USA) and Lifestream (Bard Peripheral Vascular, Tempe, AZ, USA) (18, 27). Although the internal iliac component might match better for its tapered structure, the result suggests no difference between IBDs with commercial stent or IBDs with its own bridging component in IIA exclusion. It may be due to the same distal size of these two types of bridging stent. Actually, no matter the initially devices have their own internal iliac components or require commercial stents, various bridging combinations can be used in real clinical practice to improve the applicability. In some cases, the distal landing zone can be extended into a larger IIA branch with coiling of the smaller branch simultaneously if necessary. Consequently, a short or ectatic main IIA that does not meet the anatomic criteria could be cured by IBDs in clinical practice.

The external iliac artery is considered as another essential sealing artery for iliac branch devices. As for the EIA diameter, nevertheless, the exclusion rate showed a significant difference among four IBD systems. Only one lesion was excluded by Gore for the less stringent criteria on EIA diameter (<6.5 or >25 mm). This is attributed to the GORE excluder iliac limb which could extend the Gore IBE distally into the external iliac artery. The iliac branch component alone of the IBE system can treat EIA diameters up to 13.5 mm. The external iliac artery treatment range expands to 25 mm if using one iliac extension component with a large distal size. The other three devices, however, do not contain this component in their IBD system. As a matter of fact, these three IBDs can also repair AIA with large EIA diameter by bridging commercial extension components in clinical applications. Furthermore, despite the fact that Lifetech IBSG considered the anatomical characteristics, especially CIA length and IIA diameter of the Asian population, there is no statistical significance in suitability among Lifetech and the other three devices.

Of note, our study also demonstrated a significant difference in anatomic features of patients with unilateral and bilateral lesions, especially in the diameter of dilated arteries. The average diameters of CIAs, EIAs, and IIAs, along with CIA lengths, were all unanimously larger in the bilateral group. As a result, the morphological difference excluded more bilateral lesions from the all kinds of iliac branch devices. And IIA diameter was the major reason for exclusion of bilateral lesions as well. This indicates that the affected area becomes more diffuse and extends to the IIA and EIA when bilateral CIA aneurysms are present. Concomitant bilateral CIAAs were therefore considered to be more severe, complexed anatomical and thus more difficult for treatment.

If strictly follow the IFU, the majority of the aortoiliac aneurysms are not suitable for IBD application. Pearce et al. reported that only 35% of the aneurysm repairs involving common iliac arteries would have been candidates for the Gore and Cook IBDs in western countries (5). Itoga et al. reported 17% met the criteria for Cook and 25% for Gore in Japanese (20). The inclusion percentages in our study were similar compared with the studies of Pearce et al. and Itoga et al. (5, 20). Additionally, there was no difference in suitability per patient with unilateral and bilateral lesions. This was because patients in the bilateral group were eligible for IBD on condition that one lateral lesion met the criteria. However, only 3.9% of Eastern Asian patients could be treated on both sides. Most authors advocate for the preservation of one IIA with an IBD and embolization of the contralateral IIA. However, risks of ischemic complications seem to appear in 30-55% of the patients after unilateral IIA occlusion (11). Mansour et al. reported a higher complication rate in patients with both IIA excluded compared with those preserving the contralateral one and suggested to revascularize at least one IIA in case of bilateral iliac aneurysmal involvement (28). Under this circumstance, IBDs may provide more clinical benefits for patients with bilateral iliac aneurysms. Published researches have demonstrated similar technical success and mid-term outcomes for using bilateral IBD in patients with suitable anatomical characteristics compared with unilateral iliac branched grafting (15). Few ischemic complications were reported as well in bilateral IBD implantation (29). It indicates that new generation IBDs should take bilateral lesions into consideration.

Overall, 14 lesions were barely eligible for only one IBD while 15 could be treated by all four IBDs. There were 11 lesions that only met the criteria of Gore. And overall, 32 aortoiliac aneurysms were eligible for Gore IBE which was higher than any other devices. This suggests that the Gore IBE system may be a potentially better choice for Eastern Asian patients. Nevertheless, more than half of the patients were ineligible for any iliac branch devices in this study. The IBD technology and design still require advancements, and alternative techniques for IIA preservation such as bell-bottom, sandwich and chimney techniques are continuously needed to play their role when IBDs are unsuitable in clinical practice.

There are some limitations to our study. Above all, the number of patients enrolled was still limited despite the fact that this study was based on two large tertiary centers in China, and it would be advantageous to include more patients from more centers to achieve a more solid conclusion. Besides, the evaluation of IBD suitability in these patients is purely based on anatomic criteria of the CTA results. Multiple confounding factors, such as patient general condition and medication could lead to a higher exclusion rate of IBDs.

## Conclusion

IIA preservation with any IBD was applicable for 44.7% of Eastern Asian AIA patients. IIA diameter was the main reason for failure to meet the criteria. The suitability of bilateral lesions was significantly lower than unilateral. For clinical applications on Eastern Asian patients, it is imperative to expand the IBD indications in clinical applications and evaluate the effect and efficacy in the meantime. Moreover, the design and development of next-generation IBDs should focus on internal iliac diameter suitability. In addition, the IBD system also needs to be adapted to accommodate patients with bilateral lesions in order to ensure the preservation of IIA for both sides of the patients.

## Data Availability Statement

The original contributions presented in the study are included in the article/supplementary material, further inquiries can be directed to the corresponding author/s.

## Ethics Statement

The studies involving human participants were reviewed and approved by the Institutional Review Board of Zhongshan Hospital, Shanghai (approval no. B2018-045) and Drum Tower Hospital, Nanjing (approval no. 2017-015-05). The retrospective data were anonymous, and the requirement for informed consent was therefore waived.

## Author Contributions

MZ, GW, and TY performed the measurements. EW and YFZ were involved in planning and supervised the work. ZL and LW processed the experimental data, performed the analysis, drafted the manuscript, and designed the figures. XS, YCZ, and PL performed the calculations. ZL and WF aided in interpreting the results and worked on the manuscript. All authors discussed the results and commented on the manuscript.

## Funding

This work was supported by the National Natural Science Foundation of China (grant number: 81970412), Shanghai Municipal Science and Technology Commission Innovation Fund (grant number: 18441902400), Xiamen Municipal Health Science and Technology Program Fund (grant number: 3502Z20194034), Zhongshan Hospital's Talents Supporting Plan (grant number: 2019ZSGG11), and Shanghai Engineering Research Center of Interventional Medicine (grant number: 19DZ2250300).

## Conflict of Interest

The authors declare that the research was conducted in the absence of any commercial or financial relationships that could be construed as a potential conflict of interest.

## Publisher's Note

All claims expressed in this article are solely those of the authors and do not necessarily represent those of their affiliated organizations, or those of the publisher, the editors and the reviewers. Any product that may be evaluated in this article, or claim that may be made by its manufacturer, is not guaranteed or endorsed by the publisher.
